# Efficient Stereospecific H^β2/3^ NMR Assignment Strategy for Mid-Size Proteins

**DOI:** 10.3390/magnetochemistry4020025

**Published:** 2018-06-01

**Authors:** Alexandra Born, Morkos A. Henen, Parker Nichols, Jing Wang, David N. Jones, Beat Vögeli

**Affiliations:** 1 Department of Biochemistry and Molecular Genetics, University of Colorado Anschutz Medical Campus, 12801 East 17th Avenue, Aurora, CO 80045, USA; alexandra.born@ucdenver.edu (A.B.); morkos.henen@ucdenver.edu (M.A.H.); parker.nichols@ucdenver.edu (P.N.); 2 Faculty of Pharmacy, Mansoura University, Mansoura 35516, Egypt; 3 Department of Pharmacology, University of Colorado Anschutz Medical Campus, 12801 East 17th Avenue, Aurora, CO 80045, USA; jing.5.wang@ucdenver.edu (J.W.); david.jones@ucdenver.edu (D.N.J.)

**Keywords:** NMR spectroscopy, biological macromolecules, structure calculation, stereospecific assignment, J-coupling, proteins

## Abstract

We present a strategy for stereospecific NMR assignment of H^β2^ and H^β3^ protons in mid-size proteins (~150 residues). For such proteins, resonance overlap in standard experiments is severe, thereby preventing unambiguous assignment of a large fraction of β-methylenes. To alleviate this limitation, assignment experiments may be run in high static fields, where higher decoupling power is required. Three-bond H^α^–H^β^ J-couplings (^3^*J*_Hα–Hβ_) are critical for stereospecific assignments of β-methylene protons, and for determining rotameric χ_1_ states. Therefore, we modified a pulse sequence designed to measure accurate ^3^*J*_Hα–Hβ_ couplings such that probe heating was reduced, while the decoupling performance was improved. To further increase the resolution, we applied non-uniform sampling (NUS) schemes in the indirect ^1^H and ^13^C dimensions. The approach was applied to two medium-sized proteins, odorant binding protein 22 (OBP22; 14.4 kDa) and Pin1 (18.2 kDa), at 900 MHz polarizing fields. The coupling values obtained from NUS and linear sampling were extremely well correlated. However, NUS decreased the overlap of H^β2/3^ protons, thus supplying a higher yield of extracted ^3^*J*_Hα-Hβ_ coupling values when compared with linear sampling. A similar effect could be achieved with linear prediction applied to the linearly sampled data prior to the Fourier transformation. Finally, we used ^3^*J*_Hα–Hβ_ couplings from Pin1 in combination with either conventional or exact nuclear Overhauser enhancement (eNOE) restraints to determine the stereospecific assignments of β-methylene protons. The use of eNOEs further increased the fraction of unambiguously assigned resonances when compared with procedures using conventional NOEs.

## 1. Introduction

Stereospecific assignment of methylene protons is a prerequisite for detailed structural and dynamic NMR studies of proteins [1]. Assignments of H^β2^ and H^β3^ are usually achieved from a combined use of three-bond H^α^−H^β^ J-couplings (^3^*J*_Hα−Hβ_), and nuclear Overhauser enhancement (NOE) intensities [2]. Although less common, assignments may also be supported by measurements of ^3^*J*_C’−Cγ_ [3,4] and ^3^*J*_N−Cγ_ couplings [4,5], or by estimates from chemical shifts [6]. While ^3^*J*_Hα−Hβ_ values may be extracted from ^1^H-^1^H P.E.COSY experiments for small unlabeled proteins [7], the introduction of ^13^C-isotope labeling enabled convenient three-dimensional (3D) experiments with proteins larger than 8 kDa [8,9]. However, due to the nature of methylene protons being in a similar chemical environment in proteins, there is still significant resonance overlap for H^β2^ and H^β3^ protons, in particular for proteins comprising more than 150 residues. Typically, many methylene groups cannot be stereospecifically assigned in such systems. In addition to stereospecific assignments of methylene-proton resonances in NOESY spectra, accurate measurement of ^3^*J*_Hα–Hβ_ couplings may also be directly used for χ_1_ dihedral angle determination via Karplus relationships [1].

Here, we propose an efficient strategy for stereospecific β-methylene assignment for mid-size proteins. We modified a previously described pulse sequence designed for accurate measurement of ^3^*J*_Hα–Hβ_ couplings such that a substantial increase in spectral resolution was achieved. The original pulse sequence [10] made use of continuous decoupling of ^15^N and ^13^CO which required high power when run with high static fields, leading to sample heating and possibly contributing to probe damage. Our modified version put substantially less strain on the probe. Additionally, multidimensional NMR experiments involve long measuring times since all indirect dimensions need a large number of points to be sampled. We used a 50% non-uniform sampling scheme (NUS) [11] in the indirect ^1^H and ^13^C dimensions to increase the acquisition times in the indirect dimensions without a change in measurement time. There is concern that the reconstruction of NUS data results in spectral artifacts and inaccurate relative peak intensities, which can be highly problematic for a quantitative J-coupling experiment. Furthermore, it was demonstrated that extensions of the maximum ^1^H evolution time had an increasingly reducing effect on the extracted coupling values. We demonstrated that both adverse effects were negligible for the evolution extension introduced by NUS. Finally, we combined the ^3^*J*_Hα–Hβ_ couplings with either exact nuclear Overhauser enhancement (eNOE) restraints or conventional NOEs in the assignment procedure.

We tested the strategy with two mid-size proteins, odorant binding protein 22 (OBP22) and Pin1. OBP22, from the mosquito *Aedes aegypti*, is a member of the odorant binding protein family that is implicated in mediating chemosensory signaling by transporting and presenting chemical stimuli to chemosensory receptor complexes. Previously it was shown that OBP22 expression in the salivary gland was upregulated following infection with the dengue virus, and knockdown of this OBP reduced blood-feeding behaviors of the mosquito by up to 45% when compared with control mosquitoes [12–14]. Structural studies of OBP22 using X-ray crystallography and NMR spectroscopy (Wang and Jones, unpublished) revealed that OBP22 may be involved in the recognition of bioactive lipids present on human skin. Here, we used NMR spectroscopy to determine the solution structure of OBP22 in the complex formed with arachidonic acid.

The OBP22 sample was used as our ideal case, as the protein was very well concentrated (0.7 mM), and had a fast tumbling time. We also applied this method to a less concentrated and faster-relaxing Pin1 sample. Human Pin1 is an 18.2 kDa phosphorylation dependent peptidyl-prolyl cis-trans isomerase (PPIase) that specifically isomerizes the peptide bond pS/T-P in substrates [15,16]. While Pin1 is a mitotic regulator of phosphoproteins, it is also involved in many other functions including protein folding, intracellular signaling, and transcription [17]. It is often overexpressed in cancer, yet has reduced activity in Alzheimer’s disease. Most interestingly, Pin1 is a two-domain protein that has an allosteric effector site on its N-terminal WW domain, connected to the catalytic site on the larger PPIase domain via a flexible linker [16]. Substrate binding in the high-affinity WW domain impacts the activity of the catalytic site nearly 45 Å away [18]. To study the allosteric network of Pin1, it is vital to have the greatest amount of information and resolution as possible when comparing the apo and holo structures. Therefore, it is critical to define the stereospecificity of H^β2/3^ in the side chains of Pin1. Combining these constraints with eNOEs aided in solving a high-quality, multi-state ensemble.

## 2. Experimental Section

### 2.1. Description of Pulse Sequence

**Figure 1:**
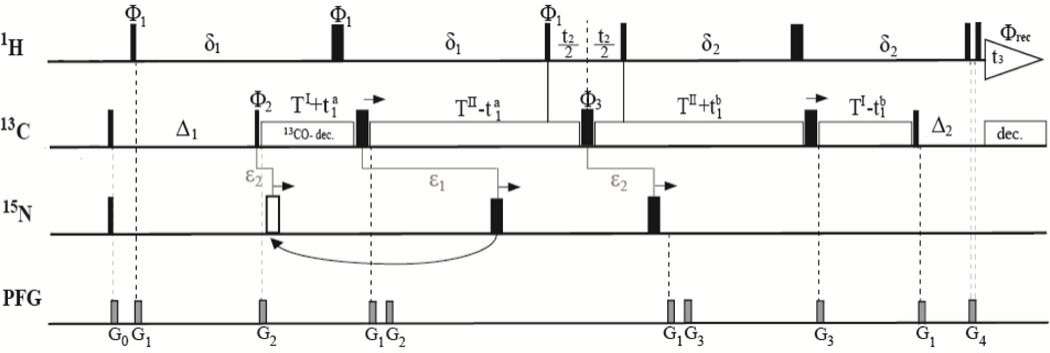
Pulse scheme for the three-dimensional (3D) HACAHB-COSY experiment for the measurement of three-bond H^α^–H^β^ J-couplings (^3^*J*_Hα–Hβ_). The ^1^H, ^13^C, and ^15^N carriers were positioned at 4.7, 46, and 119 ppm, respectively. Narrow and wide pulses denoted 90° and 180° flip angles, respectively. Delay durations were: T^I^ = 5.25 ms; T^II^ = 8.75 ms; δ_1_ = T^II^ + 2τ_90_^C^; δ_2_ = T^II^ −2τ_90_^H^; ∆_1_ = δ1 + 2τ_90_^H^ − T^I^ −τ_90_^C^ ≈ 1/(2*J*_HC_); ∆_2_ = δ2 − T^I^ − 3τ_90_^C^ ≈ 1/(2*J*_HC_), where τ_90_^H^ and τ_90_^C^ are the ^1^H and ^13^C 90° pulse lengths; *t*_1_= *t*_1_^a^ + *t*_1_^b^. ^15^N was decoupled using incremented 180° pulses. The first ^15^N pulse was applied ε_1_ = (T^I^ + T^II^)/2 = 7 ms after the first ^13^C 180° pulse if (T^I^ + T^II^)/2 < T^II^ − *t*_1_^a^/2 (black pulse), and ε_2_ = T^I^ + *t*_1_^a^/2 – 7 ms = *t*_1_^a^/2 − 1.75 ms after the ^13^C 90° pulse with phase ϕ_2_ if (T^I^ + T^II^)/2 > T^II^ − *t*_1_^a^/2 (white pulse). For ^13^CO decoupling, a SEDUCE pulse train was applied at 177 ppm [20]. Pulsed field gradients (PFG) were all sine-bell-shaped with durations of G_0_,_1,2,3,4_ = 1, 1, 0.3, 0.4, 1 ms with powers of 12, 36, 36, 20, and 16 G/cm, respectively. All pulses were applied along the x-axis unless otherwise noted. The phase cycle was: ϕ_1_ = 2x, 2(−x); ϕ_2_ = x, −x; ϕ_3_ = 4x, 4y, 4(−x), 4(−y); ϕ_receiver_ = x, −x, −x, x. Quadrature detection in *t*_1_ and *t*_2_ was obtained by incremented ϕ_2_ and ϕ_1_ using States-TPPI [21]. The pulse scheme was a modified version of the one published in Reference [10].

The pulse sequence shown in Figure 1 was a slightly modified version of the 3D HACAHB-COSY presented in Reference [10]. Due to the largely suppressed dipolar interaction between spins involved in multiple-quantum (MQ) coherence, the relaxation times of ^1^H–^13^C MQ coherences are significantly longer than those of ^1^H or ^13^C single-quantum coherence. Taking advantage of this property, the pulse sequence was essentially designed as a ^13^C^α^–^1^H^α^ constant-time HMQC (*t*_1_, *t*_3_), with a second ^1^H evolution period (*t*_2_) added in the middle of the MQ period by means of two 90° ^1^H pulses. In combination with the direct ^1^H dimension, this additional element gave rise to ^1^H–^1^H COSY-type planes. During the δ_1_ periods, ^3^*J*_Hα–Hβ_ coupling dephased H^α^ transverse magnetization and ^13^C^α^–^1^H^α^ MQ coherence such that a mixture of in-phase (2H^α^_x_C^α^_y_) and antiphase (4H^α^_y_C^α^_y_H^β^_z_) terms with respect to H^β^ were present before the 90° ^1^H pulses, marking the beginning of the *t*_2_ evolution period. Their amplitudes were cos(2π ^3^*J*_Hα–Hβ_δ_1_) and sin(2π ^3^*J*_Hα–Hβ_δ_1_), and they evolved as 2H^α^_x_C^α^_y_ and 4H^α^_z_C^α^_y_H^β^_y_ during *t*_2_, which resulted in a diagonal peak at ω_α_, and a cross peak at ω_β_ in the *t*_2_ dimension. The remainder of the pulse sequence converted the coherences back into the transverse in-phase and anti-phase H^α^ terms, adding additional factors cos(2π ^3^*J*_Hα–Hβ_δ_1_) and sin(2π ^3^*J*_Hα–Hβ_δ_1_), respectively. The peak heights of the cross peak (*I*_β_) and the diagonal (*I*_α_) were then used to determine the ^3^*J*_Hα−Hβ_ couplings:

(1) $$\frac{I_{\beta }}{I_{\alpha }}=-\tan\left(2\pi J_{\text{Hα}-\text{Hβ}}\;{\text{δ}}_{1}\right)\tan\left(2\pi J_{\text{Hα}-\text{Hβ}}\;{\text{δ}}_{2}\right)\approx -\tan^{2}\left[2\pi {\text{J}}_{\text{Hα}-\text{Hβ}}\;{\text{T}}^{\text{II}}\right].$$

The previously described pulse sequence used continuous decoupling of ^15^N and ^13^CO during the entire MQ period [10], which resulted in radio frequency (RF) heating that could alter the sample, and damage the probe. These effects are of particular importance for measurements in high magnetic fields since the required decoupling window (in units of Hertz) scales linearly with the polarizing field. In addition, the original continuous-wave ^13^CO decoupling led to a substantial Bloch–Siegert shift [10,19]. Ideally, the experiment is run on a ^13^C-only enriched sample, which renders ^15^N decoupling dispensable. However, such samples are not usually available, and typical work involves ^13^C/^15^N-labeled samples. In our modified version, ^13^CO was decoupled using a SEDUCE amplitude-modulated selective pulse scheme [20], which was recommended but not implemented in the original work [10]. ^15^N was decoupled using two incremented 180° pulses which were expected to lead to significantly reduced heating effects. The first pulse was applied during the first T^I^ + T^II^ period, and was separated by (T^I^ + T^II^)/2 (=ε_1_) from the first ^13^C 180° pulse. Initially, the decoupling pulse was placed after the ^13^C 180° pulse. However, as the ^13^C pulse was shifted to achieve *t*_1_ evolution, the decoupling pulse eventually reached the second ^1^H 90° pulse. At this point, the ^15^N decoupling pulse was placed at ε_1_, prior to the shifting ^13^C 180° pulse. This was the equivalent of applying it *t*_1_^a^/2 − 1.75 ms (=ε_2_) after the second ^13^C 90° pulse. The second ^15^N decoupling pulse was applied during the second T^I^ + T^II^ period. Because T^II^ was longer than T^I^, and the ^13^C 180° pulse was shifted to the right to achieve *t*_1_ evolution, the ^15^N pulse could be applied (T^I^ + T^II^)/2 prior to the ^13^C 180° pulse for all increments (equivalent to ε_2_ after the ^13^C 180° pulse with phase ϕ_3_), and no nonlinear repositioning was required. After processing, double quantum peaks were noticed due to the slow relaxation of methyl groups, but they did not interfere with H_a_–H_b_ cross peaks. A minimum phase cycle was used to reduce measurement time, and since the double quantum peaks were in a separate spectral region, the pulse sequence was unaltered.

### 2.2. Protein Expression and Purification

The preparation of OBP22 will be reported elsewhere. The NMR sample contained 700 µM OBP22 and arachidonic acid (1:1), in 20 mM sodium phosphate in D_2_O, with a pH of 6.5. The NMR sample was 300 µL in a standard 5 mm Shigemi tube.

Human wild-type Pin1 was cloned into pET-28a(+) (Genscript) with an N-terminal 6xhistidine tag. The protein was expressed in *Escherichia coli* strain BL21(DE3) as previously reported [22]. Briefly, overnight culture plates were resuspended in M9 minimal media enriched in ^15^N-ammonium chloride (1 g/L) and ^13^C-glucose (2 g/L) for uniform labeling. Cultures were grown at 37 °C, shaking until induction with 1 mM isopropyl-1-thio-D-galactopyranoside at optical density A_600_ = 0.8. After induction, cells were shaken at room temperature overnight. Cells were harvested by centrifugation at 4 °C for 20 min at 4000× *g*. Cells were lysed using sonication in 50 mM potassium phosphate buffer, 1 mM dithiothreitol, and 25 mM imidazole, at pH 7.5 with 0.3 mM phenylmethylsulfonyl fluoride (protease inhibitor). The cell lysate was ultracentrifuged, filtered, and then passed over a nickel-nitrilotriacetic acid column (GE Healthcare; Uppsala, Sweden) using the same buffer as above. For elution, 50 mM potassium phosphate buffer, 1 mM dithiothreitol, and 250 mM imidazole, at pH 7.5 was used. The resulting protein was concentrated and purified using Superdex 75 10/300 GL (GE Healthcare; Uppsala, Sweden) size exclusion column in 50 mM sodium phosphate buffer and 150 mM sodium chloride, at pH 6.5. The protein was concentrated with a 3000 NMWL cutoff (Millipore; Tullagreen, Ireland), and the buffer was exchanged with 20 mM sodium phosphate buffer, 50 mM sodium chloride, 5 mM dithiothreitol, and 0.03% sodium azide, at pH 6.5. The sample was then lyophilized, and resuspended in D_2_O. The NMR sample contained 300 µL of 400 µM Pin1 in a standard 5 mm Shigemi tube.

### 2.3. NMR Spectroscopy

All NMR spectra were recorded at 25 °C on a triple-resonance Varian 900 MHz spectrometer equipped with a cryo-probe. The linear sampling spectra of OBP22 and Pin1 were acquired with 1024 complex points in the direct ^1^H dimension, and 56 and 72 complex points in the indirect ^1^H and ^13^C dimensions, respectively. Non-uniform sampling schemes were generated by the NUS@HMS generator software [11], with 1024 complex data points in the direct ^1^H dimension, and 50% sampling of the original 89 and 90 complex points in the ^1^H and ^13^C dimension, respectively, resulting in the same overall measurement times as those for linear sampling. The spectral widths for both the linear and NUS sampling spectra were 14,044.9 Hz (direct ^1^H), 10,793.3 Hz (indirect ^1^H), and 6785.4 (^13^C) with an interscan delay of 1.6 s, and eight scans. Linear prediction was performed on noted linearly sampled data with the order set to 28 in the indirect ^1^H dimension. The NUS-acquired data were reconstructed using the hmsIST software [11]. Zero filling was achieved by rounding the size to a power of two (auto) in both indirect dimensions. Both linear and NUS sampling experiments took 60 h for measurement.

### 2.4. J-Coupling Value Determination and Stereospecific Assignment Protocol

After processing and visualizing the data in NMRPipe [23], spectra were analyzed and assigned using CCPNMR [24]. Cross-peak H^α^ and diagonal H^β^ peaks that did not significantly overlap were picked for each residue. When H^β^ peaks were partially overlapped, but it was clear which species had the stronger intensity (trans position), the values were still used. As reported in the original ^3^*J*_Hα–Hβ_ coupling publication, low dispersion of H^α^ peaks contributed to partial overlap, which made peak volumes not as reliable as intensities measured using peak heights [10]. Even missing H^α^–H^β^ cross peaks, indicating small ^3^*J*_Hα–Hβ_ values consistent with a gauche conformation, were still useful in determining the stereospecificity of methylene protons. In that case, we used the noise threshold for the cross-peak intensity, which effectively set an upper limit to the extracted coupling value. Most of the gauche H^α^–H^β^ cross peaks in the OBP22 spectra were above the noise level, unlike in the case of Pin1, due to lower protein concentration, and the larger overall tumbling time of the latter. The experimental ^3^*J*_Hα–Hβ_ coupling values were calculated using Equation (1), and the associated error considering the spectral noise threshold (NT) was calculated by the following equation:

(2) $$\sigma_{J_{H_{\alpha }-H_{\beta }}}=\frac{\text{NT}\sqrt{-\frac{I_{\beta }}{I_{\alpha }}-\frac{I_{\alpha }}{I_{\beta }}}}{4{\text{πT}}^{\text{II}}\left(I_{\alpha }-I_{\beta }\right)}.$$

For 33 protons, the ^3^*J*_Hα–Hβ_ coupling could only be determined from one experiment in the Pin1 sample. For the 98 protons with couplings able to be extracted accurately from both experiments, the values were averaged for the subsequent protein structure calculation in CYANA. For the OBP22 sample, all 117 values were able to be extracted accurately from both experiments, and as such, the values were also averaged. The propagated error for these averaged values was based on the individual, original errors calculated for NUS (*σ*_*N*_) and the linear sampling (*σ*_*L*_) methods by Equation (3).

(3) $$\sigma_{NL}=\frac{\sqrt{\sigma_{N}{}^{2}+\sigma_{L}{}^{2}}}{2}.$$

To determine the stereospecific assignment of the H^β^ protons, and the rotameric conformations of Pin1, these ^3^*J*_Hα–Hβ_ values were used in combination with ^3^*J*_HN–Hα_ couplings, and either conventional NOEs or eNOEs, following a procedure similar to the one described in Reference [25]. Conventional NOEs could also be used for this assignment protocol instead of eNOEs, although the yield of stereospecific constraints would be reduced owing to less precise distance restraints [25]. For OBP22, the stereospecific assignment was performed using only conventional NOEs with the extracted ^3^*J*_Hα–Hβ_ values. An additional error of 1 Hz was added to the ^3^*J*_HN–Hα_ and ^3^*J*_Hα–Hβ_ couplings to account for systematic errors common to both sampling schemes and approximations in the Karplus parameterization. In brief, these restraints were then used in a first structure calculation in CYANA [26], followed by a second calculation after swapping the two methylene H^β^ protons (“stereo.cya script”). If the target function was reduced by a user-defined input threshold value (i.e., 0.1 Å^2^), or more for one conformation over another, the methylene protons would, thus, be stereospecifically assigned. After the first run of this stereo-assignment script, these newfound assignments were used as additional fixed restraints to run another structure calculation, reducing the target function. Then, the calculation was run again using the same constraints as the first time, except for the new structural coordinates, as well as the previously found stereo-assignments. This whole procedure was run iteratively until no new stereo-specific assignments were identified. To test the impact in the stereospecific yield due to the ^3^*J*_Hα–Hβ_ values and eNOEs, Pin1 was evaluated in four cases: either with conventional NOEs or eNOEs, and with or without the ^3^*J*_Hα–Hβ_ couplings.

## 3. Results and Discussion

### 3.1. Comparing NUS and Linear Sampling ^3^J_Hα–Hβ_ Values in OBP22

For the comparison of the linear and NUS sampling schemes, we recorded the HACAHB-COSY with identical overall running times, but sampled only 50% of the points, resulting in ca. 60% and 25% extensions of the maximum evolution times in the indirect ^1^H and ^13^C dimensions, respectively. Note that the resulting resolution in the indirect ^1^H dimension was still lower than the 33 Hz by which the peaks were split due to ^1^*J*_Cα–Cβ_ splitting.

**Figure 2:**
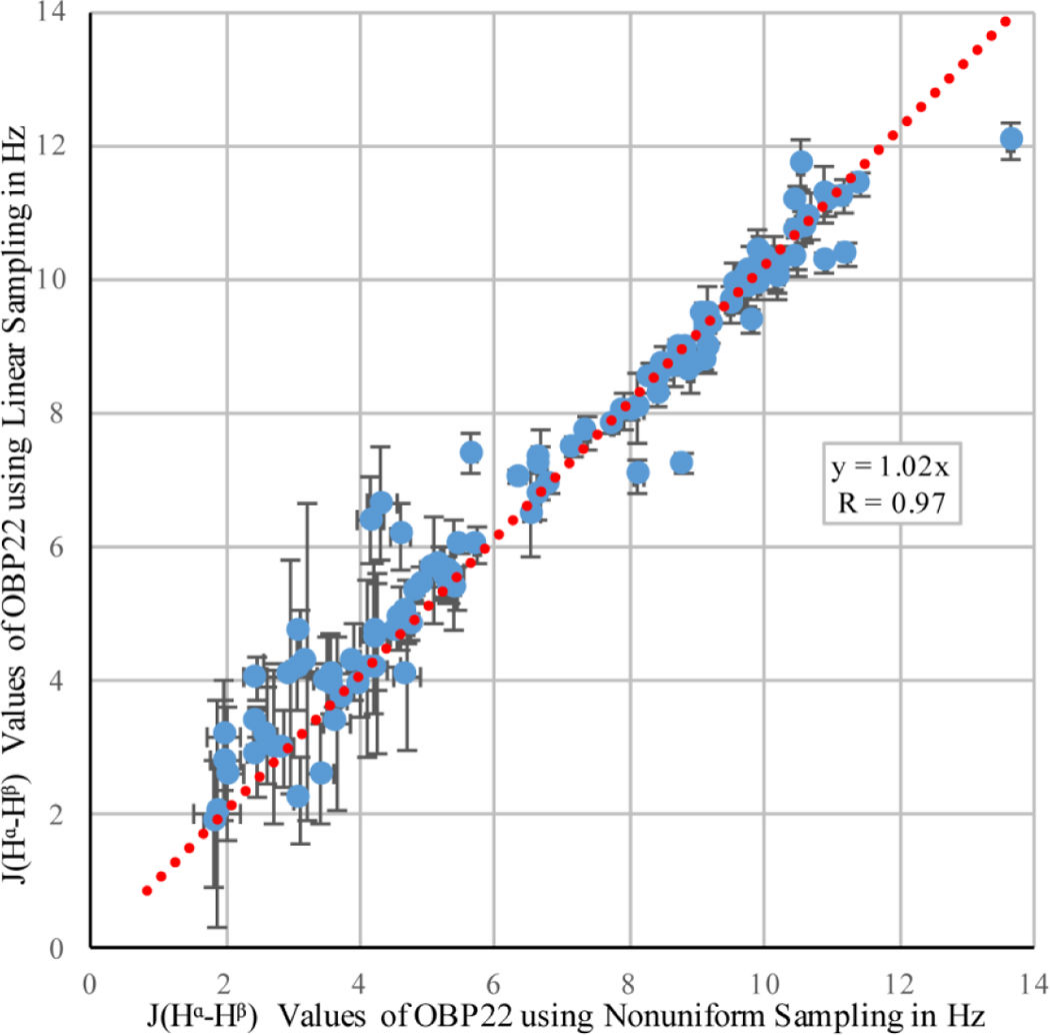
Correlation between ^3^*J*_Hα–Hβ_ coupling values in odorant binding protein 22 (OBP22) obtained from non-uniform (NUS) and linear sampling. The error bars were calculated using Equation (2).

In order to reduce random errors that potentially masked the comparison of the two sampling schemes, we chose an OBP22 sample that was nearly twice as concentrated as the Pin1 sample, and had a shorter overall tumbling time (7 ns for OBP22 versus 13 ns for Pin1), allowing the low-intensity, gauche configuration peaks to be above the noise level. All 117 couplings were measured in both experiments, and the correlation of the values is plotted in Figure 2.

Pearson’s correlation coefficient, *R* = 0.97, demonstrated that the linear and NUS sampling methods were in very good agreement. The experimental errors calculated from the spectral noise were 0.34 Hz and 0.23 Hz. Based on these errors, the expected difference between the values from both sampling schemes was 0.41 Hz if no systematic error was introduced by either of the two methods. This was indeed the case, as the root-mean-square deviation (RMSD) between the two datasets was nearly identical (0.43 Hz).

It was pointed out that the differential autorelaxation, and scalar coupling network, active during the indirect ^1^H evolution period of the diagonal and cross peaks, resulted in an underestimation of the true coupling values when the peak intensities were used instead of the volumes [10]. For a protein the size of OBP22, the cross peaks were attenuated by ca. 1%–5%, resulting in 1%–3% underestimation of the extracted couplings. This effect was enhanced upon increasing maximum ^1^H evolution periods. In the presented implementations, the period used for linear sampling was 5.2 ms, and was extended to 8.2 ms for NUS. This potentially increased the relative attenuation of the cross peak by a further 3%, and the corresponding couplings were potentially underestimated by up to 5%. A second source contributing to the underestimation of the true coupling was the differential autorelaxation of the in-phase and antiphase terms with respect to H^β^ during the T^I^ + T^II^ periods [27,28]. The effect was proportional to the overall tumbling time, and in the presented case, underestimated the trans coupling values by 15%–20%, while the gauche values were negligibly affected. This effect was independent of the sampling scheme, and therefore, did not affect the comparison between the linear and NUS datasets. The slope of the linear regression through the correlation plot in Figure 2 was 1.02, indicating that coupling values extracted from NUS were 2% smaller than those from the linear sampling scheme. This reduction agreed well with the theoretical prediction outlined above. However, this value was well within experimental uncertainty, and we cannot be certain that it was caused by increased maximum evolution time. In conclusion, our findings indicated that the non-uniform sampling with its subsequent reconstruction did not introduce any artefacts in the relative peak intensities.

### 3.2. Comparing NUS and Linear Sampling ^3^J_Hα–Hβ_ Values in Pin1

**Figure 3:**
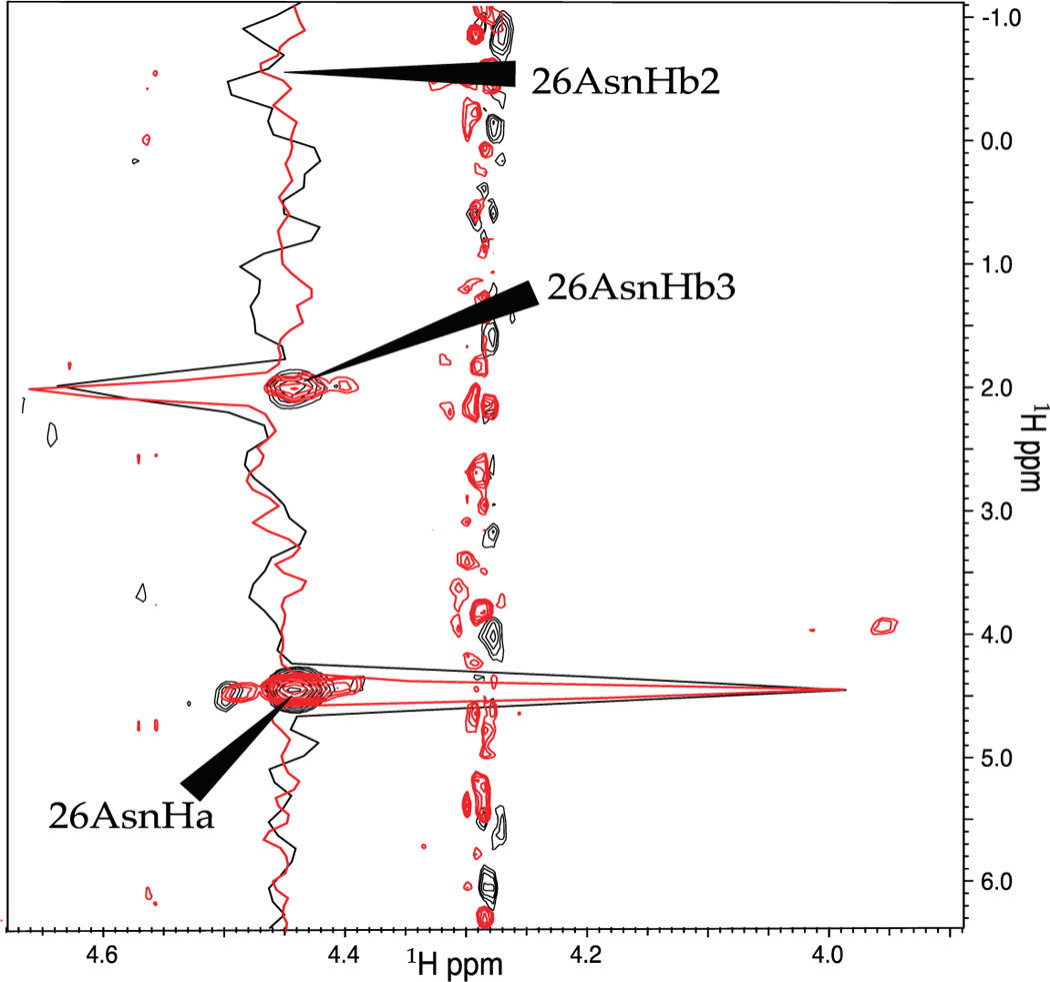
Comparison of spectra recorded with linear and NUS sampling. Shown are contour plots and slices through the peaks of Asn 26. The linearly sampled and the NUS spectra are colored black and red, respectively. The slices are scaled such that the diagonal peaks have equal intensity.

**Figure 4:**
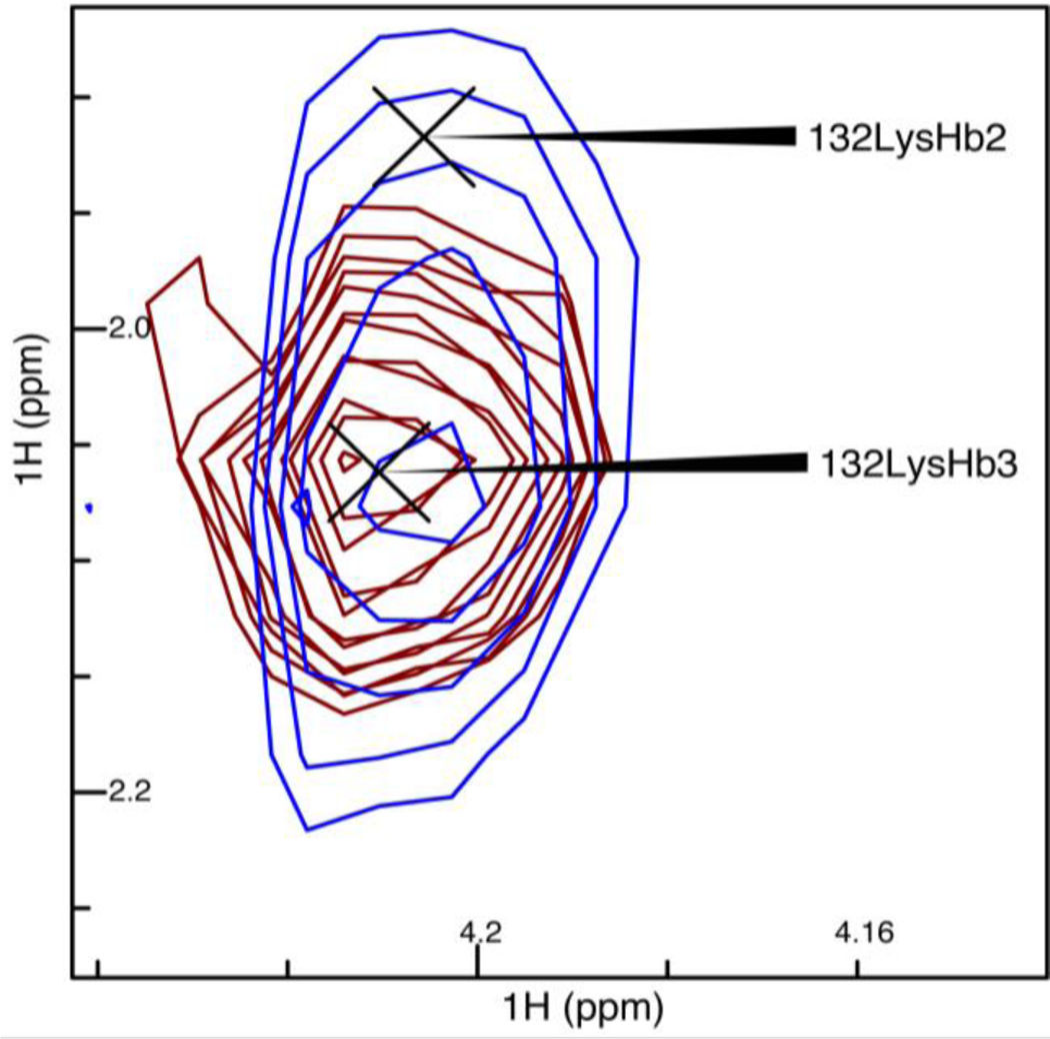
Spectral overlay of linear sampling versus NUS for ^3^*J*_Hα–Hβ_ in blue and red, respectively. Shown are the cross peaks of lysine at position 132 (Lys132). The spectra had a base level normalized to the noise level of the spectra, and had 20 levels with the level multiplier set to 1.2.

Next, we repeated the comparison of the linear and NUS sampling schemes with Pin1, which was nearly half as concentrated as OBP22, and had a larger overall tumbling time. The narrowing of the peaks when NUS was employed was exemplified by the peaks from residue 26 in Figure 3. Although there was no rigorous way to compare the noise of the spectra due to the noise being unevenly distributed in NUS spectra, it appeared that there was less noise in the region surrounding these particular peaks in the NUS spectrum. After deletion of the ^3^*J*_Hα–Hβ_ values for 132 methylene-peak pairs from the linearly sampled experiment due to overlap between H^β2^ and H^β3^, the experiment yielded 103 ^3^*J*_Hα–Hβ_ couplings, while the NUS experiment resolved 126 couplings. An example where the NUS scheme resolved the methylene peaks that were overlapped in the spectra obtained from linear sampling is shown in Figure 4. Only inspection of the NUS spectrum revealed that the true height of the cross peak at 1.9 ppm in the indirect dimension vanished in the noise, while the broader linewidth of the strong cross peak at 2.05 ppm caused the intensity to appear above noise level.

**Figure 5:**
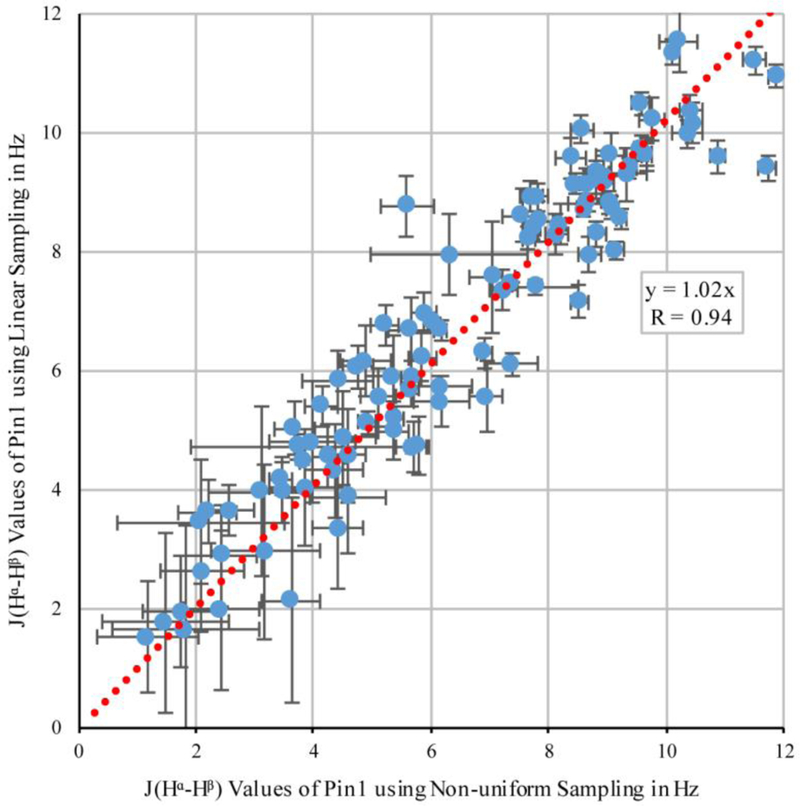
Correlation between ^3^*J*_Hα–Hβ_ coupling values in Pin1 obtained from non-uniform and linear sampling. The error bars were calculated using Equation (2).

Figure 5 shows a correlation plot of the couplings that were determined in both experiments. Despite the lower signal-to-noise ratio than that obtained for OBP22, the correlation between the values was still high (*R* = 0.94). The experimental error calculated from the spectral noise was 0.6 Hz for both sampling schemes. Based on these errors, the expected difference between the values from the different sampling schemes was 0.8 Hz if no systematic error was introduced by either of the two methods. This was indeed the case, as the root-mean-square deviation between the two datasets was only slightly larger (1.0 Hz).

Exactly like the correlation observed for OBP22, the slope of the linear regression was 1.02, indicating that coupling values extracted from NUS were also 2% smaller than those from the linear sampling scheme. Again, this correlation agreed well with the prediction that the NUS-value reduction was caused by increased evolution time; however, this was also potential experimental uncertainty. In conclusion, the Pin1 data confirmed the findings for OBP22 that non-uniform sampling did not substantially impact the relative peak intensities.

As in the prior work [10], J_αβ_ couplings were evaluated for alanines to identify any systematic reductions in the measured J-couplings from the ideal value of 7 Hz. The average J_αβ_ coupling for alanine in the NUS Pin1 dataset was 6.8 ± 0.7 Hz, while the average value for the linear Pin1 set was 7.2 ± 0.9 Hz. While the previous report noticed a reduction in values due to a faster relaxation of antiphase terms, both linear and NUS data for Pin1 did not significantly deviate from 7 Hz. This independently confirmed that we did not introduce a systematic shift in the measured values.

In addition, we tested whether linear prediction of the linearly sampled data prior to Fourier transformation resulted in a similar yield and quality of the extracted couplings. We were able to evaluate the same number of couplings as with NUS. The relative peak intensities were also approximately conserved, resulting in a similar correlation to that observed for NUS data, when compared to couplings extracted from linear sampling without linear prediction (Supplementary Figure S1 , *R* = 0.94). Supplementary Figure S2 shows a correlation plot of NUS and linear sampling with linear prediction, again demonstrating a high correlation. Taken together, the data suggested that linear prediction and NUS did not disrupt the relative peak intensities. Since the comparison between linear sampling and NUS yielded the same correlation as that between the two linear sampling approaches, despite being based on two independent raw data-sets, we recommend the use of NUS rather than linear prediction for linearly sampled data.

### 3.3. Stereospecific Assignment Using CYANA

For both OBP22 and Pin1, ^3^*J*_Hα–Hβ_-coupling-constant files (.cco) were made for input into CYANA, with Karplus curves set to A = 7.23, B = −1.37, and C = 2.40 [29]. The target function cutoff for the stereospecific-assignment swapping in CYANA was the preset 0.1 Å^2^. Note that this value had the potential to be optimized.

A structure calculation for OBP22 with the ^3^*J*_Hα–Hβ_ couplings and conventional NOEs was performed with subsequent stereo-assignment. As summarized in Table 1, 39 stereospecific assignments were found, all of which were methylene protons. When the structure calculation and protocol were run with only NOEs, no stereospecific assignments were found. Using this protocol with OBP22, 42.4% of methylene protons were stereospecifically assigned.

Identical to OBP22 when only using conventional NOEs as restraints, no stereospecific assignments were found for Pin1. When ^3^*J*_Hα–Hβ_ couplings were added into the calculation, the iterative protocol yielded 66 stereospecific assignments. Next, we converted Pin1 conventional NOEs into eNOEs using our recently developed buildup series method to see if more precise distance restraints would impact the yield of stereospecific assignments [30]. Indeed, eNOEs were extremely helpful in defining rotameric states and bond angles, even without the ^3^*J*_Hα–Hβ_ couplings. A modest increase in the number of stereospecific assignments was made when the couplings were added into the calculation. The 114 stereospecific residues found using eNOEs and ^3^*J*_Hα–Hβ_ couplings are listed in Supplementary Table S1. Due to less precise distance restraints in conventional NOEs, the ^3^*J*_Hα–Hβ_ couplings made a much larger impact on the stereospecificity of Pin1 using only conventional NOEs, when compared with the eNOE calculations. While all three assignment results for Pin1 had slightly different residues that were determined to be stereospecifically defined, there was no case of an H^β2^/H^β3^ pair being swapped from one assignment to another. Using eNOEs in this protocol for Pin1, 45% of methylene protons were stereospecifically assigned. Overall, it was evident that eNOEs were extremely valuable for defining the stereospecificity of the protein.

**Figure 6:**
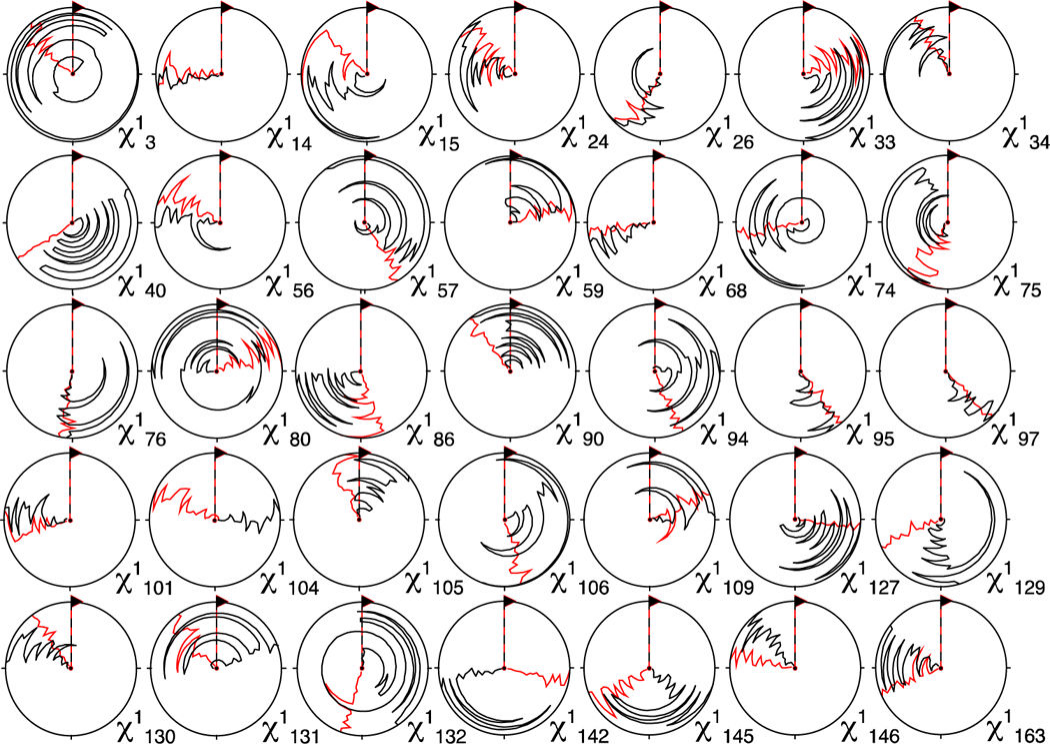
Circle plots of χ_1_ angles of stereospecifically assigned methylene protons using the method presented (**red**). Shown are the residues for which the angle distributions were narrowed the most when compared with angles in the structures calculated from exact nuclear Overhauser enhancements (eNOEs) and J-couplings without stereospecific assignment (**black**). The 20 conformations with the lowest CYANA target function were used. A similar plot depicting all residues is presented in Figure S3 in the Supporting Information.

An immediate assessment of the impact of the stereospecific assignment could be obtained from analysis of the resulting χ_1_ dihedral angles. Figure 6 shows circle plots depicting the χ_1_ angles of the 20 CYANA Pin1 structures with the lowest target-functions on a per-residue basis. The angle distributions were generally substantially narrower when the J-couplings and eNOE restraints were used in combination with stereospecific assignment than without such an assignment (in that case the CYANA command “distances stereoexpand” had to be used). Interestingly, a similar narrowing effect was also observed for many residues without assignment, or even without J-couplings (see Figure S3 in the Supplementary Information). Apparently, better definition of the assigned methylenes propagated into neighboring residues.

As expected, the stereospecific assignment also resulted in lower RMSD values of structural bundles obtained from the CYANA structure calculation, which were indicative of a higher precision of the resulting structures (see Table 2). When applied to the eNOE/J-coupling restraints, the improvement in the backbone of Pin1 was 0.48 Å and 0.21 Å for the WW domain and the PPIase domain, respectively, and 0.52 Å and 0.29 Å for all heavy atoms. When the conventional NOE/J-coupling restraints were stereospecifically assigned, the drop in RMSD was still 0.16 Å and 0.25 Å for the backbone, and 0.26 Å and 0.28 Å for all heavy atoms for the WW domain and the PPIase domain, respectively. Interestingly, omission of the scalar couplings resulted in similar RMSD values to those when used without stereospecific assignment. This was due to the CYANA command “distances stereoexpand” effectively averaging the restraints over the two methylene protons. The RMSD from the X-ray structure with Protein Data Bank (PDB) accession code 1pin dropped substantially when adding J-couplings to the eNOE input dataset, but without stereospecific assignments (see Table 2). It dropped further when the stereospecific assignment was used. This trend was not observed in the same analysis of structures calculated with conventional NOEs. We note that, while we are confident about the overall chemical shift assignment, we are currently establishing the ideal eNOE restraint dataset for eNOE-based multi-state ensemble calculations. This involves a critical assessment of long-range restraints in particular. At this point, our preliminary eNOE structures were calculated with all eNOE restraints extracted from reasonable buildup curves. For this reason, the RMSD of the eNOE structure was larger than that of the conventional NOE structure. We could not exclude that the larger RMSDs was also caused in part by a lower density of the eNOEs than of the conventional NOEs in specific segments of Pin1. However, the stereospecific assignment was primarily driven by the short-range NOE data, which we do not expect to be altered in the future restraint assessment, thus already enabling a higher yield of stereospecific assignments for the eNOE structures at this stage.

### 3.4. Correlation between ^3^J_Hα–Hβ_ Couplings in Pin1’s WW Domain to Isolated WW Domain

**Figure 7:**
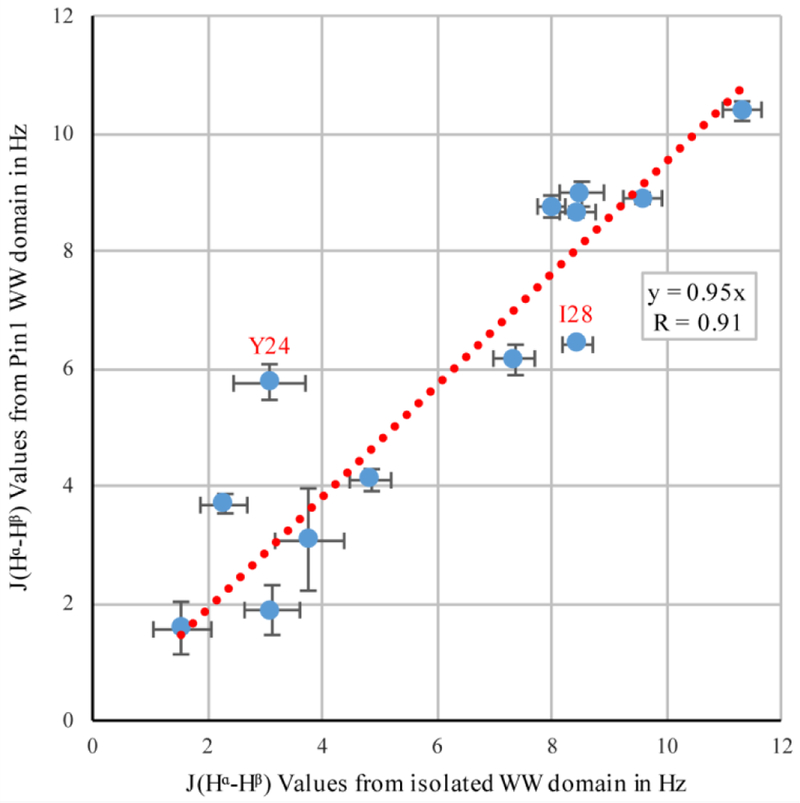
Correlation between ^3^*J*_Hα–Hβ_ coupling values in the isolated WW domain, and the WW domain of full-length Pin1. For the isolated WW domain, the originally published pulse sequence [6] was used, whereas the full-length Pin1 was evaluated in this study. The two strongest outliers are highlighted.

As mentioned earlier, Pin1 has a WW domain in addition to its PPIase catalytic domain, and these two domains are connected via a flexible linker [7]. ^3^*J*_Hα–Hβ_ couplings were previously determined for the isolated 34-residue WW domain, using the originally proposed pulse sequence [10] with linear sampling [31]. Figure 7 shows a comparison of these previously determined isolated WW-domain values with the couplings we detected in the WW domain of full-length Pin1. The generally high correlation (*R* = 0.91) indicated that the side-chain rotamers in the WW domain were not substantially altered by the presence of the PPIase domain.

The isolated WW domain had two mutations to help its stability, a tyrosine to phenylalanine at position 23 (Y23F), and a tryptophan to phenylalanine at position 34 (W34F), while the full-length Pin1 in this study was wild type. While the mutant residues of the isolated WW domain were not compared to the residues of the Pin1 WW, the largest deviation came from residue Y24, which was directly next to one of the mutations. The next largest deviation came from residue isoleucine 28 (I28), which is a key residue in the interdomain interface in full-length Pin1 [32]. Residues Y24 and I28 were in slightly different environments in the two proteins, which could explain the slight deviations in their coupling values.

## 4. Conclusions

In summary, we tested an efficient strategy for the assignment of H^β2^ and H^β3^ chemical shifts for proteins larger than 150 residues. We modified a ^3^*J*_Hα–Hβ_ HACAHB-COSY with favorable relaxation properties, such that heating caused by extensive decoupling was not prohibitive in high magnetic fields (900 MHz in the presented application). We demonstrated that the improved resolution afforded by high fields can be further increased by non-linear sampling of both indirect dimensions. We applied the approach to two mid-size proteins, and showed that the NUS artefacts did not lead to measurable errors in the extracted ^3^*J*_Hα–Hβ_ coupling values. When combined with eNOEs, the dataset yielded stereospecific assignment for a large fraction of the β-methylene protons.

## Supplementary Material

Supplemental_Data Figure S1: Correlation between linear sampling only and linear sampling with linear prediction for ^3^*J*_Hα–Hβ_ coupling values in Pin1; Figure S2: Correlation between non-uniform sampling and linear sampling with linear prediction for ^3^*J*_Hα–Hβ_ coupling values in Pin1; Figure S3: Circle plots showing χ_1_ angles for the 20 CYANA Pin1 structures with the lowest target-functions on a per-residue basis. Angle distributions of structures calculated from eNOE and J-couplings without stereospecific assignment are in black, while those from stereospecifically assigned eNOEs and J-couplings are in red. Circle plots in blue are residues which were found to be stereospecifically assigned in our protocol; Table S1: Stereospecific assignments found in Pin1 using eNOEs and ^3^*J*_Hα–Hβ_ coupling values.

## Figures and Tables

**Table 1.** Number of stereospecific assignments determined using the presented pulse sequence paired with CYANA calculations.

Restraints Used	Number Total Stereospecific Assignments	Number H_B2/3_ Stereospecific Assignments Only
OBP22 NOEs alone	0	0
OBP22 NOEs + ^3^*J*_Hα-Hβ_ couplings	39	39
Pin1 NOEs alone	0	0
Pin1 NOEs + ^3^*J*_Hα-Hβ_ couplings	66	42
Pin1 eNOEs alone	103	48
Pin1 eNOEs + ^3^*J*_Hα-Hβ_ couplings	114	54

**Table 2.** Root-mean-square deviations (RMSDs) for various structure calculations for the ensemble compared with its average, as well as with the crystal structure (PDB code 1pin) [16].

Restraints Used	RMSD [Å], Backbone	RMSD [Å], Heavy Atom	RMSD to 1pin [Å], Backbone (Heavy Atom)
Pin1 eNOEs alone	WW: 2.12 ± 0.54 PPIase: 1.26 ± 0.17	WW: 2.93 ± 0.53 PPIase: 1.81 ± 0.13	WW: 1.69 (2.31) PPIase: 2.27 (3.16)
Pin1 eNOEs + ^3^*J*_Hα-Hβ_ couplings, not ass.	WW: 1.95 ± 0.85 PPIase: 1.28 ± 0.21	WW: 2.66 ± 0.84 PPIase: 1.88 ± 0.24	WW: 1.32 (2.15) PPIase: 2.43 (3.19)
Pin1 eNOEs + ^3^*J*_Hα-Hβ_ couplings, ass.	WW: 1.47 ± 0.43 PPIase: 1.07 ± 0.14	WW: 2.12 ± 0.47 PPIase: 1.59 ± 0.16	WW: 1.19 (2.04) PPIase: 1.90 (2.72)
Pin1 NOEs alone	WW: 0.72 ± 0.20 PPIase: 0.84 ± 0.08	WW: 1.21 ± 0.24 PPIase: 1.37 ± 0.1	WW: 1.44 (2.46) PPIase: 2.26 (2.89)
Pin1 NOEs + ^3^*J*_Hα-Hβ_ couplings, not ass.	WW: 0.75 ± 0.24 PPIase: 1.03 ± 0.16	WW: 1.30 ± 0.27 PPIase: 1.52 ± 0.15	WW: 1.55 (2.57) PPIase: 2.27 (2.88)
Pin1 NOEs + ^3^*J*_Hα-Hβ_ couplings, ass.	WW: 0.59 ± 0.29 PPIase: 0.78 ± 0.11	WW: 1.04 ± 0.28 PPIase: 1.24 ± 0.11	WW: 1.47 (2.24) PPIase: 2.32 (3.08)
