# Supplementary material for: Efficient Stereospecific Hβ2/3 NMR Assignment Strategy for Mid-Size Proteins
Source: Magnetochemistry. Author manuscript; Available in PMC 2019 May 13. (PMC6513325; doi:10.3390/magnetochemistry4020025)
Supplement: Supplemental_Data — Figure S1: Correlation between linear sampling only and linear sampling with linear prediction for 3JHα–Hβ coupling values in Pin1; Figure S2: Correlation between non-uniform sampling and linear sampling with linear prediction for 3JHα–Hβ coupling values in Pin1; Figure S3: Circle plots showing χ1 angles for the 20 CYANA Pin1 structures with the lowest target-functions on a per-residue basis. Angle distributions of structures calculated from eNOE and J-couplings without stereospecific assignment are in black, while those from stereospecifically assigned eNOEs and J-couplings are in red. Circle plots in blue are residues which were found to be stereospecifically assigned in our protocol; Table S1: Stereospecific assignments found in Pin1 using eNOEs and 3JHα–Hβ coupling values. [file NIHMS1023867-supplement-Supplemental_Data.pdf]

Supplementary Materials

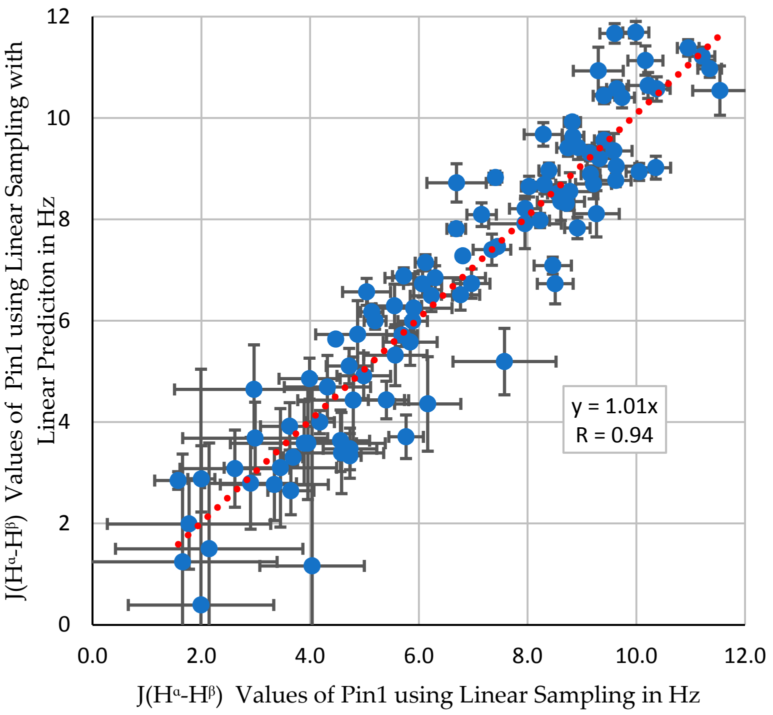

Figure S1. Correlation between linear sampling only and linear sampling with linear prediction for $^3J_{H\alpha-H\beta}$ coupling values in Pin1.

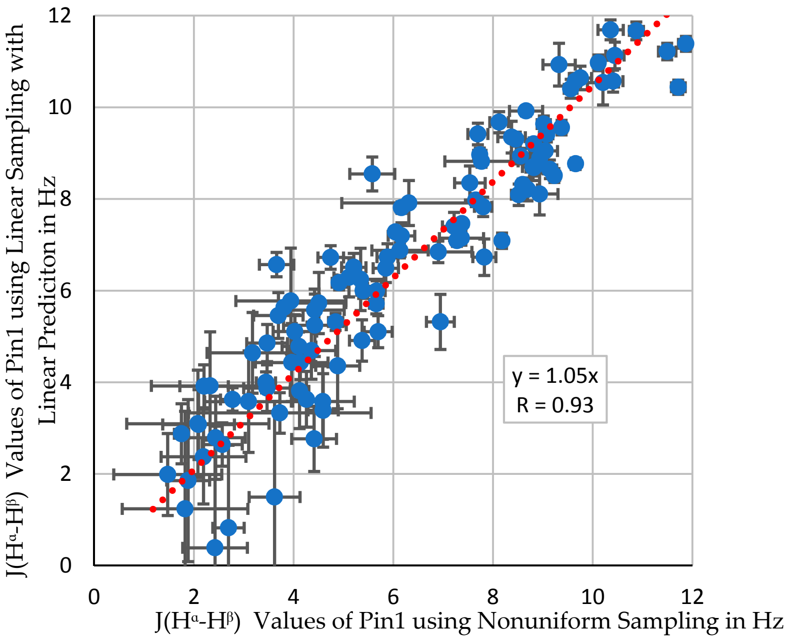

Figure S2. Correlation between non-uniform sampling and linear sampling with linear prediction for $^3J_{H\alpha-H\beta}$ coupling values in Pin1.

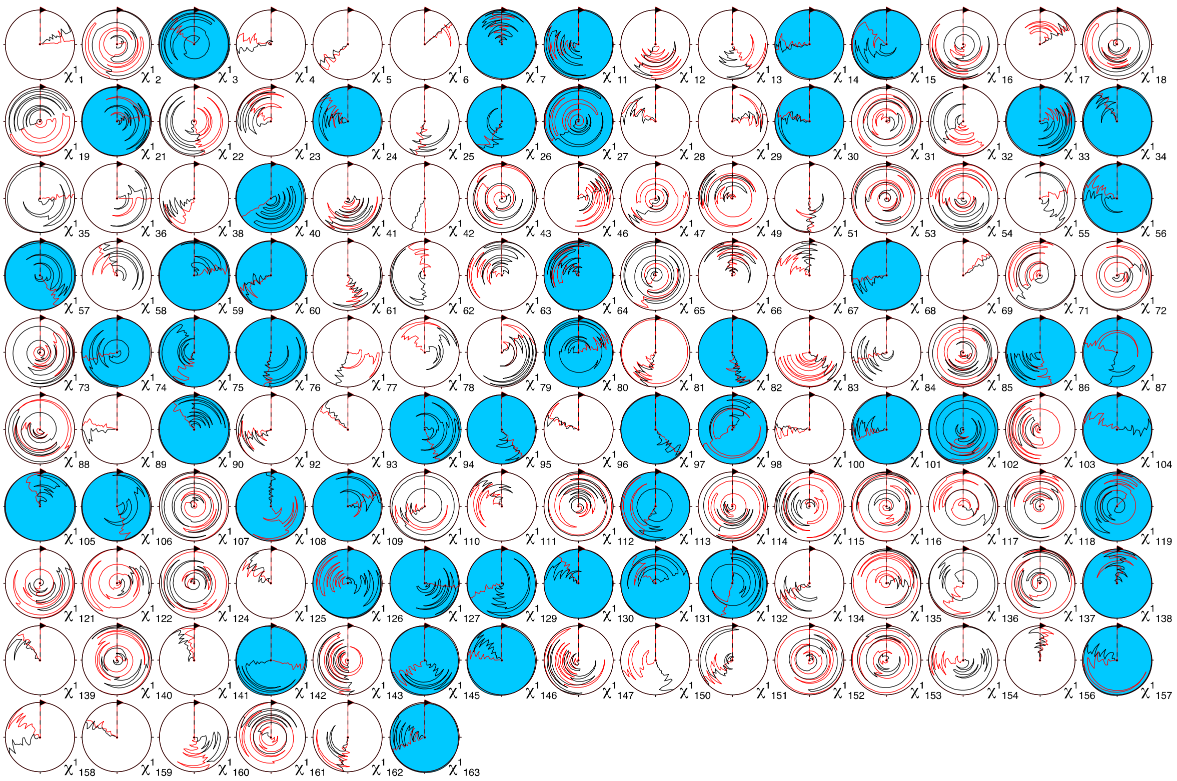

Figure S3. Circle plots showing χ_1 angles for the 20 CYANA Pin1 structures with the lowest target-functions on a per-residue basis. Angle distributions of structures calculated from eNOE and J-couplings without stereospecific assignment are in black, while those from stereospecifically assigned eNOEs and J-couplings are in red. Circle plots in blue are residues which were found to be stereospecifically assigned in our protocol.

Table S1. Stereospecific assignments found in Pin1 using eNOEs and $^3J_{H\alpha\text{–}H\beta}$ coupling values.

#	Residue	Stereoassigned 1	Chemical Shift 1 (ppm)	Stereoassigned 2	Chemical Shift 2 (ppm)
1	MET	HG2	2.428	HG3	2.492
3	ASP	HB2	2.607	HB3	2.694
6	LYS	HG2	1.384	HG3	1.402
6	LYS	HD2	1.658	HD3	1.683
6	LYS	HE2	2.969	HE3	3.004
7	LEU	HB2	1.798	HB3	1.389
7	LEU	QD1	1.063	QD2	0.777
9	PRO	HD2	3.897	HD3	3.627
11	TRP	HB2	3.241	HB3	2.948
14	ARG	HB2	0.108	HB3	1.26
15	MET	HB2	1.819	HB3	1.846
15	MET	HG2	2.395	HG3	2.238
20	GLY	HA2	4.081	HA3	3.903
21	ARG	HB2	1.718	HB3	1.956
22	VAL	QG1	1.009	QG2	0.777
24	TYR	HB2	2.647	HB3	2.885
26	ASN	HB2	-0.701	HB3	1.983
27	HIS	HB2	3.071	HB3	3.364
28	ILE	HG12	0.924	HG13	1.223
30	ASN	HB2	2.89	HB3	3.108
30	ASN	HD21	6.792	HD22	7.537
33	GLN	HB2	3.836	HB3	3.749

33	GLN	HE21	6.596	HE22	7.377
34	TRP	HB2	3.593	HB3	3.171
36	ARG	HG2	1.189	HG3	0.956
36	ARG	HD2	2.991	HD3	3.024
40	ASN	HB2	2.823	HB3	2.736
40	ASN	HD21	6.935	HD22	7.599
46	LYS	HG2	1.414	HG3	1.37
52	PRO	HB2	2.287	HB3	1.994
55	VAL	QG1	0.064	QG2	-0.627
56	ARG	HB2	1.702	HB3	1.248
56	ARG	HG2	1.163	HG3	1.391
56	ARG	HD2	2.912	HD3	2.869
57	CYS	HB2	3.116	HB3	2.852
59	HIS	HB2	3.636	HB3	3.318
60	LEU	HB2	1.517	HB3	1.034
60	LEU	QD1	0.572	QD2	0.146
61	LEU	QD1	-0.291	QD2	0.678
62	VAL	QG1	0.945	QG2	1.166
63	LYS	HD2	1.474	HD3	1.915
64	HIS	HB2	3.126	HB3	2.696
68	ARG	HB2	1.786	HB3	1.922
68	ARG	HG2	1.582	HG3	1.778
70	PRO	HB2	2.571	HB3	1.849
74	ARG	HB2	0.669	HB3	0.945
74	ARG	HG2	-0.027	HG3	-0.645
75	GLN	HB2	1.727	HB3	1.659
75	GLN	HG2	2.027	HG3	2.076
76	GLU	HB2	1.956	HB3	2.025
77	LYS	HG2	1.188	HG3	1.33
78	ILE	HG12	1.096	HG13	1.184
80	ARG	HB2	2.023	HB3	1.956
80	ARG	HG2	1.619	HG3	1.446
82	LYS	HB2	1.341	HB3	1.075
82	LYS	HG2	0.766	HG3	1.013
83	GLU	HG2	2.201	HG3	2.427
86	LEU	HB2	2.006	HB3	1.426
86	LEU	QD1	1.001	QD2	1.069
87	GLU	HB2	2.019	HB3	2.06
87	GLU	HG2	2.177	HG3	2.293
88	LEU	QD1	0.632	QD2	0.8
89	ILE	HG12	1.51	HG13	1.183
90	ASN	HB2	2.775	HB3	2.644
90	ASN	HD21	6.576	HD22	7.281
93	ILE	HG12	2.101	HG13	1.009
94	GLN	HB2	2.262	HB3	2.214
94	GLN	HE21	6.885	HE22	7.462
95	LYS	HB2	2.115	HB3	1.656
95	LYS	HG2	1.718	HG3	1.559
95	LYS	HD2	1.704	HD3	1.565
96	ILE	HG12	0.648	HG13	1.807
97	LYS	HB2	1.949	HB3	1.9
97	LYS	HG2	1.729	HG3	1.498
98	SER	HB2	4.123	HB3	4.058
101	GLU	HB2	1.965	HB3	1.347
102	ASP	HB2	2.921	HB3	2.684
104	GLU	HB2	2.219	HB3	2.006
105	SER	HB2	3.923	HB3	3.988

106	LEU	HB2	1.467	HB3	0.712
106	LEU	QD1	0.653	QD2	0.724
108	SER	HB2	3.929	HB3	3.841
109	GLN	HB2	1.068	HB3	0.767
109	GLN	HG2	1.628	HG3	2.102
109	GLN	HE21	6.711	HE22	6.954
113	CYS	HB2	2.777	HB3	2.522
117	LYS	HG2	1.167	HG3	1.226
117	LYS	HE2	2.458	HE3	2.638
119	ARG	HB2	2.141	HB3	2.014
126	SER	HB2	4.073	HB3	3.971
127	ARG	HB2	1.591	HB3	1.51
127	ARG	HG2	0.59	HG3	1.031
127	ARG	HD2	2.717	HD3	2.668
129	GLN	HB2	2.28	HB3	2.107
129	GLN	HG2	2.369	HG3	2.295
129	GLN	HE21	7.55	HE22	6.99
130	MET	HB2	1.8	HB3	1.683
131	GLN	HB2	2.295	HB3	1.928
132	LYS	HB2	1.933	HB3	2.065
132	LYS	HG2	1.464	HG3	1.514
132	LYS	HD2	1.625	HD3	1.77
138	SER	HB2	3.802	HB3	3.524
141	LEU	QD1	0.768	QD2	0.537
142	ARG	HB2	1.868	HB3	1.565
144	GLY	HA2	3.634	HA3	4.211
145	GLU	HB2	2.239	HB3	1.933
146	MET	HB2	2.164	HB3	1.792
146	MET	HG2	2.327	HG3	3.063
150	VAL	QG1	0.902	QG2	0.687
156	ILE	HG12	1.616	HG13	0.813
158	ILE	HG12	1.867	HG13	0.948
160	LEU	QD1	0.442	QD2	0.315
163	GLU	HB2	1.732	HB3	1.939

© 2018 by the authors. Submitted for possible open access publication under the terms and conditions of the Creative Commons Attribution (CC BY) license (http://creativecommons.org/licenses/by/4.0/).
